# A Nutritional Bioenergetic Model for Farmed Fish: Effects of Food Composition on Growth, Oxygen Consumption and Waste Production

**DOI:** 10.1155/anu/9010939

**Published:** 2025-07-26

**Authors:** Orestis Stavrakidis-Zachou, Ep H. Eding, Nikos Papandroulakis, Konstadia Lika

**Affiliations:** ^1^Institute of Marine Biology, Biotechnology and Aquaculture, Hellenic Centre for Marine Research, Heraklion, Greece; ^2^Aquaculture and Fisheries Group, Wageningen University and Research, Wageningen, Netherlands; ^3^Department of Biology, University of Crete, Heraklion, Greece

**Keywords:** DEB theory, digestion, nutritional model, *Oncorhynchus mykiss*, oxygen consumption, protein-to-energy ratio, total ammonia production

## Abstract

The study of flow and transformation of energy and nutrients via mathematical modelling provides an in silico tool approach for designing scientific experiments, improving precision in aquaculture production and reducing the need for experimental animals. The proposed nutritional bioenergetics model is based on the dynamic energy budget (DEB) theory, a mechanistic framework to study individual metabolism. The model is an extension of the typical DEB models in that it includes a digestion module where the protein and non-protein food components contribute to assimilation via the concept of a synthesising unit (SU). The model allows predictions for measurable quantities of interest for aquaculture, including feeding rate, oxygen consumption, carbon dioxide, ammonia and solid waste production, under various temperatures and feeding conditions, both in terms of quantity and macronutrient composition. The feeding schedule's effects, such as the diurnal variation in waste production in response to feeding frequency, are also captured. The model quantifies the effects of the dietary protein-to-energy ratio on food intake and assimilation; energy-rich diets or those with excessive or poor amounts of protein show reduced intake. The model has been parametrised and validated for rainbow trout (*Oncorhynchus mykiss*) to demonstrate its capabilities. Testing the model with diverse datasets has shown that it predicts weight gain well, and to a lesser extent, oxygen consumption and total ammonia production. The proposed model could be a useful in silico tool for fish researchers, technicians and farm operators.

## 1. Introduction

Finfish aquaculture is the fastest-growing agricultural sector and is of great importance for food security and access to high-quality animal protein which is needed to cover the demands of the increasing human population [1]. A key element for the successful and financially viable operation of fish farms is the quantification and forecasting of fish growth and metabolism under different rearing environments. Specifically, knowledge of how environmental factors as well as husbandry parameters affect fish growth performance is of prime interest for the industry [2]. While the former refers to parameters such as the temperature, oxygen content, pH and salinity of the water the latter relate to the operations that take place in the farm including the density at which fish are stocked, the feeding schedule and the nutritional content of the offered feeds (here, denoting food specifically designed for livestock). Nutrition in particular is a research field of huge interest not only because it plays a critical role in fish growth and health as well as production costs but also because it influences waste production which in turn determines the ecological footprint of fish farming on the environment [3]. For instance, nitrogen derived from protein catabolism is one of the main nutrients for water pollution in aquaculture facilities, while solid and chemical wastes also play a role in the degradation of the surrounding environments [4, 5]. With regard to fish nutrition, the chemical composition of the feed greatly influences the processes of ingestion, digestion and assimilation of energy [6, 7]. Fish have species-specific dietary requirements for various macronutrients present in their feeds, with particular emphasis often placed on the protein-to-energy ratio, receiving the highest attention in nutritional studies [8, 9]. Such requirements differ both across species and developmental stages thus constituting an area of active nutritional research not only for emerging species due to the diversification of aquaculture but also for established ones, particularly for the larval and broodstock stages, which are the most poorly understood [10]. In recent years, the definition of requirements has expanded to include amino acid, fatty acid, vitamin and mineral requirements while there is a growing need to also define them under cases of stress either environmental (such as temperature and hypoxia) or due to pathogens [11].

Apart from defining the nutritional requirements, important aspects of research nutrition entail feed management, evaluation of new ingredients and the formulation of diets that not only meet production goals but also promote the health and welfare of fish (functional feeds). In particular, the increasing need to replace fishmeal and oil with alternative sources has been one of the fastest growing fields of aquaculture research, assessing the performance of plant-based products, and lately other sources like insects, microalgae, yeasts and bacteria, targeting to environmental and climate sustainability while applying concepts of circular bioeconomy [10, 11]. The evaluation of these new diets in terms of palatability, digestibility, utilisation, nutrient interaction, interaction with gut microbiota and influence on immunology and health will become increasingly important. Additionally, the assessment of food safety risks of aquaculture products due to the use of bioproducts in feedstuff [12], the use of GMO and the potential reduction in their nutritional value is crucial, especially since most of the new ingredients lack the omega 3 and long-chain polyunsaturated fatty acid levels of traditional fish feed sources [13]. Feed management plays a critical role in reducing wastage and increasing production efficiency, which is why the increase in automation and adoption of new technologies (such as models and artificial intelligence) over the past decade is only expected to increase in the future [11].

Elucidation of the species-specific dietary requirements, as well as the effects of important environmental factors on fish metabolism, often requires conducting physiological trials under controlled laboratory conditions. Indeed, the main goal of many experiments with fish is to investigate the assimilation of energy via feeding and its subsequent partitioning into various metabolic processes, which is the field of quantitative bioenergetics [8, 14–16]. While laboratory experiments provide critical data, they are often resource-intensive and may involve procedures that can affect animal welfare, such as blood sampling, injections, tagging or surgical interventions, potentially impacting both ethical standards and data quality [17–19]. In accordance with the 3Rs, introduced over 60 years ago and now integrated innational legislation in many countries [20], alternative approaches such as the use of in vitro and in silico testing [21] are increasingly incorporated into fish research to complement in vivo studies.

In recent years, the aquaculture industry has been increasingly incorporating precision farming tools such as mathematical models, artificial intelligence, automation and robotics [22, 23]. These tools aim to improve the production process, increase the yield and decrease the associated costs as well as ensure the welfare of the farmed animals. In that respect, nutritional models play an integral part in fish farming since they provide effective implementation of feed and management of the production process by improving the feed efficiency, reducing waste output and producing less feed waste [24, 25]. Traditionally, fish models in aquaculture have predominantly been statistical ones. These models rely on statistical regressions conducted on collected empirical data (experiments or in situ), while they are often highly accurate. Their applicability is limited to the system under investigation [26]. Traditional bioenergetic models [27] including those based on the factorial approach [14, 15] as well as their extensions such as the Energy and Protein flux model [28] also find wide use in fish nutrition research and offer advantages due to their ability to achieve a high degree of realism based on limited sets of parametrisation data. However, for maximising the capacity of mathematical models to reduce the need for the use of laboratory animals, they need to be more general and encompass a wider set of applications. A solution to that could be the use of mechanistic models as much as possible, which has been advocated by many researchers [26, 29–31]. These models describe cause–effect relationships over time, and in the case of fish, they typically incorporate the underlying physiological processes that govern the bioenergetics of fish.

In this article, we have developed a nutritional bioenergetic model for aquaculture fish, addressing industry needs for improved planning and design of fish trials, precision in production and reduced reliance on experimental animals through an in silico tool approach, providing an alternative to traditional fish trials. This mathematical tool aims to describing the bioenergetics of fish in relation to growth, oxygen consumption and waste production in response to feeding in terms of feeding level, feed composition and feeding frequency. To accomplish that, we use the dynamic energy budget (DEB) theory of metabolic organisation, which utilises established physiological and thermodynamic mechanisms to specify the acquisition and allocation of energy within an individual throughout its life cycle [32]. This framework has been widely implemented in fish research and especially in the field of aquaculture [31, 33–35] and was chosen here particularly due to its mechanistic nature and high capacity to describe cause–effects relationships; the need for which has been highlighted earlier. However, current applications focus predominantly on the effects of environmental conditions under the assumptions of fixed food stoichiometry and constant feeding levels, thus posing limitations in their use as tools for nutritional research. In this context, our work aims at explicitly modelling food dynamics by incorporating a digestion-assimilation module while accounting for varied feed composition at the macronutrient level as well as feeding frequency. To showcase the capabilities of the presented modelling approach, we use rainbow trout (*Onchorhyncus mykiss*) as our species of study. The species selection was spurred not only by its importance as an established, commercially farmed species but also by the rich literature archive, which provides sufficient data for parametrisation, validation and demonstration purposes.

## 2. Model Description

### 2.1. Model Structure

The nutritional bioenergetic model is based on an ensemble of rules that describe the processes of digestion, absorption and the allocation of energy derived from food to metabolic processes of growth, maintenance, maturation and reproduction according to a set of priority rules. The model is an extension of the standard DEB model and assumes three life stages (larvae, juvenile and adult) as well as metabolic accelerated development for early stages, which is an established practice for studying ray-finned fish species in DEB context [36–38]. The most important transitions include ‘birth', which is marked by the start of exogenous feeding, ‘metamorphosis' as the completeness of metamorphosis and ‘puberty', denoted by developmental completeness and the start of allocation to reproduction. This approach allows for the tracking of individual fish metabolism through all stages relevant to aquaculture, which may not completely overlap with the aforementioned DEB life stages. For instance, the on-growing stage of production usually contains fish that transition from the juvenile to adult stages before reaching harvest size. Life stage transitions occur when the cumulative investment into maturation reaches certain thresholds. Additionally, the model incorporates a digestion–assimilation module to simulate the food dynamics in the alimentary track and the process of assimilation of the macronutrients from the gut wall.

The state of an individual fish is described by four variables: volume of structural mass *V*, energy reserve *E*, energy invested in maturation *E*_*H*_ and energy invested in reproduction *E*_*R*_ (for adults). Structure, reserve and reproduction reserve contribute to organism's biomass, and maturity tracks the developmental transitions between life stages. The state variables, the energy fluxes and the dynamics of an individual fish are summarised in Table S1 of the Supporting Information (Section S.1). For a more comprehensive description of the DEB theory and a full list of the equations and the nomenclature used, see Kooijman [32] and Stavrakidis-Zachou et al. [39]. The stomach, which is treated as an 'environment' for the fish, is used to smooth out fluctuations in food availability, which is usually provided at regular intervals. The derivation of the equations is given in the following section.

One of the core assumptions of DEB theory is that all organic compounds, food, faeces, structural mass and reserves (the latter two comprise the individual biomass), consist of a mixture of polymers such as proteins, lipids and carbohydrates which form generalised compounds of constant chemical composition. The composition of a generalised compound is expressed as the relative abundance of hydrogen (H), oxygen (O) and nitrogen (N) to carbon (C). Thus, for example, a molecule of reserve has the formula *CH*_*n*_HE__*O*_*n*_OE__*N*_*n*_NE__, where *n*⁣_⁎__*E*_ are the chemical indices, for example, *n*_*NE*_ represents the molar N:C ratio of reserve. Each generalised compound has a specified chemical potential (*μ⁣_⁎_*), specific density (*d⁣_⁎_*) and molecular weight (*w⁣_⁎_*). Therefore, identification of the chemical indices of the mineral and organic compounds found in the food, the structure and the reserves allows for the quantitative and qualitative assessment of metabolic waste output under various experimental scenarios. While multiple reserve systems have been established for autotrophs [40, 41], DEB models for animals are generally built upon the assumption of one type of food, one reserve and one structure, with the exception of a two-reserve model published recently for hares [42]. In this study, we propose an extension to the model by incorporating the concept that food is comprised of 'two substrates', each with a different composition. Specifically, we assume that a fraction of food is protein, while the remaining is composed of lipids and carbohydrates. As a result, the food is digested into two separate compounds: protein and non-protein macronutrients. This differentiation is useful as it enables us to trace the fate of nitrogenous waste via the digestion of the protein component, while the non-protein digestion, which is based on the digestibility of lipids and carbohydrates, is conducted separately; ash is not explicitly considered in the dynamics of food and reserve, but it is traced to the faeces. The derivation of the chemical indices for all organic compounds is provided in the Supporting Information (Section S.3).

**Figure 1:**
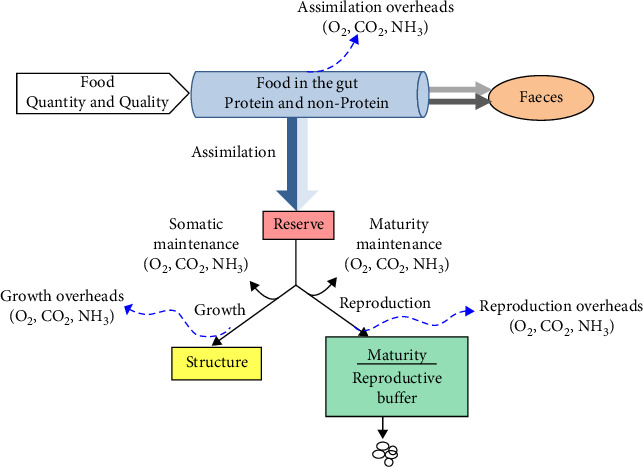
Conceptual representation of the metabolic processes. Food is partitioned into protein and non-protein components. Energy assimilated from food is added into reserves and subsequently allocated to fuel the metabolic processes: a fixed fraction *κ* of the mobilised flux is allocated to somatic maintenance and growth and the remaining (1−*κ*) to increase and maintain maturity or to reproduction. This representation is an extension of the standard DEB conceptualisation in that it includes the digestion of food in the gut and the partitioning of food into protein and non-protein components.

A schematic representation of the model is shown in Figure 1. Incoming food, which is partitioned into protein and non-protein components, enters the alimentary track where the process of digestion takes place. Food uptake depends on food availability and fish size. Ingested food is first converted into reserves (a process called assimilation) with a constant efficiency, which is specific to food composition. Mobilised reserves are thereafter allocated to somatic and maturity maintenance, growth (i.e., increase in structural body mass) and maturation/reproduction. A fixed fraction *κ* of the mobilised energy is used for somatic functions, such as somatic maintenance and growth, while the remaining 1-*κ* fraction is allocated to maturation/reproduction, after subtraction of maturity maintenance costs.

### 2.2. Digestion–Assimilation Module

The alimentary track of the digestive system of fish, including mouth, stomach, intestines, liver and pancreas, plays a vital role in digestion which is the process of modifying the feed macronutrients into molecules and elements so that they can be assimilated through the intestinal wall [43]. To do that, apart from mechanical puncturing, crushing and grinding of the food, digestive enzymes and other components are secreted, which break down the food polymers and aid in their absorption. For simplicity, we will not distinguish between the various parts of the alimentary track and from now on we will refer to it in its entirety as stomach. Enzymes attack food particles, break them down and generate products that will then be absorbed through the digestive wall and form the generalised reserve molecules. The rate at which the products are generated by the digestion process is proportional to the digestive surface area, which determines the enzymic secretions [44] and the mass of food, *M*_*X*_ (in mol), in the stomach. This rate therefore is noted as ${\dot{J}}_{d}=\left\{{\dot{J}}_{Xgm}\right\}L^{2}M_{X}$, where $\left\{{\dot{J}}_{Xgm}\right\}$ is the maximum surface-specific rate of digestion and *L* the structural length, a metric for the size of the individual (see Supporting Information, Section S.2 for the derivation).

Suppose that a fraction *a*_*P*_ of the food in the stomach is protein and the remaining 1 − *a*_*P*_ non-protein. The absorption of the products through the digestive wall and the transformation into reserves (assimilation process) is modelled using the synthesising unit (SU) concept of DEB theory [32]. The SUs are generalised enzymes that bind and process one or more substrates to form one or more products. The two complementary substrates protein, *X*_*P*_, and non-protein, *X*_*nP*_, are processed in parallel to produce the generalised reserves, *E*. The rate of reserve formation or assimilation rate is

(1) $$\begin{array}{c} {\dot{J}}_{\text{EA}}=\left\{{\dot{J}}_{{\text{EA}}_{m}}^{d}\right\}f_{X}L^{2}, \end{array}$$

where *f*_*X*_ represents the scaled functional response for digestion given by $f_{X}=\frac{M_{X}}{M_{X}+M_{K}^{X}}$, with

(2) $$\begin{array}{c} M_{K}^{X}=\frac{\left\{{\dot{J}}_{{\text{EA}}_{m}}^{d}\right\}}{\left\{{\dot{J}}_{Xg_{m}}\right\}}\;\left({\left(y_{{\text{EX}}_{P}}a_{P}\right)}^{-1}+{\left(y_{{\text{EX}}_{nP}}\left(1-a_{P}\right)\right)}^{-1}-{\left(y_{{\text{EX}}_{P}}a_{P}+y_{{\text{EX}}_{nP}}\left(1-a_{P}\right)\right)}^{-1}\right). \end{array}$$

$\left\{{\dot{J}}_{{EA}_{m}}^{d}\right\}$ and $\left\{{\dot{J}}_{Xg_{m}}\right\}$ are, respectively, the surface-specific maximum assimilation and digestion rates. *y*_EX_*P*__ and *y*_EX_*nP*__ are, respectively, the molecules of protein and non-protein substrates required to form a molecule of reserve. The derivation of Equations (1) and (2) is given in the Supporting Information (Section, S.2).

The rates at which the protein and non-protein parts of food are used to form reserves (in mol/d), respectively,

(3) $$\begin{array}{c} {\dot{J}}_{X_{P}}^{+}=\left\{{\dot{J}}_{{\text{EA}}_{m}}^{d}\right\}f_{X}L^{2}/y_{{\text{EX}}_{P}}\text{and }{\dot{J}}_{X_{nP}}^{+}=\left\{{\dot{J}}_{{\text{EA}}_{m}}^{d}\right\}f_{X}L^{2}/y_{{\text{EX}}_{nP}}. \end{array}$$

The rate of faeces production (${\dot{J}}_{P}$, mol/d) is proportional to reserve formation rate Equation (1) and is equal to

(4) $$\begin{array}{c} {\dot{J}}_{P}=y_{PE}\left\{{\dot{J}}_{EA_{m}}^{d}\right\}f_{X}L^{2}, \end{array}$$

where *y*_PE_ is the yield of faeces on reserve (mol *P*/mol *E*).

### 2.3. Dynamics of Food in Stomach

The food uptake in fish is regulated by interactions between the brain and hormonal factors that control appetite [45]. For aquaculture in particular, food is delivered by automatic feeders or by hand at a frequency and quantity according to the farmers' feeding plan [46]. Depending on the species, the life stage and temperature, several meals may be provided within the day, which typically last a few minutes followed by longer periods of digestion, while the provided quantities are carefully regulated to prevent food spillage [4, 47]. Here, we will assume that the duration of food uptake is short (minutes) compared to the digestion process, which begins at feeding but lasts up to several days. In other words, we do not explicitly model the feeding process but rather assume an instantaneous increase of food in the stomach upon the ingestion of the food. This simplification allows the model to handle different feeding strategies with ease, ranging from constant feeding to starvation.

The maximum stomach capacity, measured as dry mass content of stomach, *M*_gm_, (in mol) is assumed to be proportional to the structure of the animal and is written as [*M*_gm_]*V*, where [*M*_gm_] (mol/cm^3^) is the volume-specific maximum stomach capacity for food. The stomach capacity depends on its geometry as well as the type of food (energy content, density, etc.), since the same stomach volume translates to different amounts of mass for foods of different composition. If fish is fed in meals as fraction, *k*_*X*_, of their body (wet) weight, the amount of food (g) given per meal is *k*_*X*_*W*. To convert it into (ash-free) dry mass, it must be multiplied by the ratio of dry-to-wet mass of food $\frac{d_{Xd}}{d_{Xw}}$, where *d*_*Xd*_ and *d*_*Xw*_ are, respectively, the specific densities of dry and wet food (g/cm^3^) and divide by the molecular weight of dry food *w*_*X*_ (g/mol) to convert in moles. The ingested food is based on the minimum between the deficit of the stomach (i.e., the 'stomach-space' that can still be filled in) and the food provided. If *M*_*X*_ is the (ash-free) dry mass of food in the stomach in moles, the amount of dry mass of food (in mol) consumed per meal (*i*) is given by

(5) $$\begin{array}{c} {M_{X}}_{i}=\min\left(\left[M_{\text{gm}}\right]V-M_{X},\;k_{X}\frac{d_{Xd}}{d_{Xw}}\frac{W}{w_{X}}\right). \end{array}$$

Between meals, the rate of change in stomach content is given by

(6) $$\begin{array}{c} \frac{d}{dt}M_{X}=-\left({\dot{J}}_{X_{P}}^{+}+{\dot{J}}_{X_{nP}}^{+}+{\dot{J}}_{P}\right)=-\left(y_{X_{P}E}+y_{X_{nP}E}+y_{\text{PE}}\right)\left\{{\dot{J}}_{{\text{EA}}_{m}}^{d}\right\}fL^{2}, \end{array}$$

where the first two terms represent the food absorbed by the digestive wall to form reserves (Equation (3)) and the third term that lost as faeces (Equation (4)).

### 2.4. Product Formation and Other Measurable Quantities

Mass fluxes of organic (food, faeces, reserves and structure) and non-organic, also referred as mineral in the DEB context, (O_2_, CO_2_, nitrogenous waste) compounds can be written as weighted sum of three basic fluxes (in J/day): assimilation (${\dot{p}}_{A}$), growth (${\dot{p}}_{G}$) and dissipation (${\dot{p}}_{D}$) (Table S1, Supporting Information). Dissipation represents metabolic work that converts reserve into mineral products in ways that do not lead to the production of new biological material, that is, work due to maintenance, maturation (for larvae and juveniles) and reproduction overheads (for reproductive adults) (for details see Kooijman [32] and Stavrakidis-Zachou et al. [39]).

The rate of oxygen consumption, the non-faecal nitrogenous waste and the carbon dioxide production rates are, respectively, ${\dot{J}}_{O}=\eta_{OA}{\dot{p}}_{A}+\eta_{OD}{\dot{p}}_{D}+\eta_{OG}{\dot{p}}_{G}$, ${\dot{J}}_{N}=\eta_{ND}{\dot{p}}_{D}+\eta_{NG}{\dot{p}}_{G}$ and ${\dot{J}}_{C}=\eta_{CA}{\dot{p}}_{A}+\eta_{CD}{\dot{p}}_{D}+\eta_{CG}{\dot{p}}_{G}$. The weight coefficients follow from the mass balance of the elements C, H, O and N (Chapter 4.3 in Kooijman [32]). The nitrogen lost via faeces amounts to ${\dot{J}}_{PN}=14n_{NP}\frac{y_{PE}}{\mu_{E}}{\dot{p}}_{A}$, where *n*_NP_ the molar N : C ratio of faeces.

Body mass of an individual has contributions from structure (*V*), reserve (*E*) and (for reproducing adults) energy reserve for reproduction (*E*_*R*_). Mass quantified as wet weight is given by

(7) $$\begin{array}{c} W=d_{Vw}\left(V+\left(E+E_{R}\right)\frac{w_{Ed}}{d_{Ed}\mu_{E}}\right), \end{array}$$

where *w*_*Ed*_ is the molecular weight of dry reserve (g/mol), *μ*_*E*_ the chemical potential of reserve (J/mol) and *d*_*Vw*_ and *d*_*Ed*_ are, respectively, the specific densities of wet structure and dry reserves (g/cm^3^) (for details see Section 3.2.1 in Kooijman [32]). Since the gut is conceptually treated as a tube with open ends passing through the animal, both ingested food and faeces are considered part of the external environment for the animal and are not included in the calculation of the organism's body mass. This may introduce small deviations when comparing DEB predictions to isolated weight measurements that include gut contents. However, the effect is negligible when analysing growth over time, as the contribution of transient gut content averages out across time-series data. Stomach volume *V*_*g*_ relates to structural volume *V* with the shape coefficient *δ*_*g*_ : *V*_*g*_ = *δ*_*g*_*V*. Water balance plays a role in digestion when fish are fed a dry diet [48, 49], which should be considered when calculating the maximum storage capacity. Apart from a small amount of water included in the food fed, some water is also absorbed by the food before ingestion, while water is also supplied by the stomach and the proximal part of the intestine during digestion [50]. If for each gram of dry food fed, *y*_*HX*_*d*__ grams of water is required in the stomach to moisturise the food, the volume occupied by *M*_*X*_ dry mass of food in the stomach amounts to $\frac{y_{HX_{d}}w_{X}M_{X}\;}{d_{H}}+\frac{w_{X}M_{X}\;}{d_{Xd}}$ and should not exceed the stomach volume *V*_*g*_. Thus, at the maximum capacity, it holds that

(8) $$\begin{array}{c} V_{g}=\frac{y_{HX_{d}}w_{X}M_{\text{gm}}}{d_{H}}+\frac{w_{X}M_{\text{gm}}}{d_{Xd}}, \end{array}$$

where the first term corresponds to the volume occupied by the absorbed water (with *d*_*H*_ = 1 g/cm^3^ the density of water) and the second term is the volume occupied by the dry mass of the food. Solving Equation (8) for the maximum dry mass content of stomach *M*_*gm*_, we obtain $M_{gm}={\left(y_{HX_{d}}\frac{d_{Xd}}{d_{H}}+1\right)}^{-1}\frac{d_{Xd}}{w_{X}}\delta_{g}V$, from which we deduce that the volume-specific maximum stomach capacity for food is $\left[M_{gm}\right]=\;{\left(y_{HX_{d}}\frac{d_{Xd}}{d_{H}}+1\right)}^{-1}\frac{d_{Xd}}{w_{X}}\delta_{g}$.

### 2.5. Data for Model Calibration and Validation

For any given fish species, the DEB parameters can be either estimated [39, 51] using the freely downloadable DEB tool software (http://www.bio.vu.nl/thb/deb/deblab/) and a number of zero- and univariate data sets, or be retrieved from the AmP collection (https://www.bio.vu.nl/thb/deb/deblab/add_my_pet/). The AmP database has parameter estimates for 913 species in the class of Actinopterygii including many of the major farmed species. In the present study, we apply our model to rainbow trout (*Oncorhynchus mykiss*). The DEB parameter values were retrieved from the AmP database. The add-on module for digestion was calibrated using additional data on gastric evacuation at different temperatures and fish size [52], stomach volume at different fish weights [49, 53], as well as data relating to the water content and dry mass content of the stomach [49], which are shown in 3.1. The methodological details for these datasets including the size and number of fish, the temperature and the data collection procedures are provided in the Supporting Information (S.1).

For model validation, data on weight, oxygen consumption carbon dioxide production and total ammonia nitrogen (TAN) excretion from different studies were used. The datasets included different fish sizes, temperatures and diets that differed in quantity as well as quality. Specifically, both published [54–58] and unpublished (INRAE NuMéA) sources, with trials using different diets, rearing protocols and starting fish weights were used to evaluate the performance of the model with respect to its growth predictions. In total, seven growth datasets were evaluated, three of which involves fish from experimental trials, while the other four involved fish reared in a commercial setting either in cages or in flow-through raceways. The inclusion of the commercial data was done in order to demonstrate the performance of the model under actual farming conditions and widen its applicability. For the gaseous exchange, oxygen consumption and carbon dioxide production measurements were obtained from literature under different temperatures, fish sizes and feeding conditions ranging from fasted to ad libitum fed fish [59–61]. Similarly, published data regarding TAN excretion levels were obtained for different feeding rates including fasted and well-fed fish [60, 62]. For fasted fish, we imposed an empty stomach, effectively removing assimilation from the model dynamics, which otherwise remained as described in Table S1, Supporting Information. Because the duration of fasting was short, we did not implement additional starvation rules (like shrinking or rejuvenation). The methodological details for each of the aforementioned datasets including the type of trial, the culture system, the size of the fish, the feeding regime (level, schedule, composition), the temperature, the region of study and the specific variable measured are summarised in the Supporting Information (Table S4).

The validation data were taken from tables from their respective publications or in the case of figures, digitised with PlotReader (v1.56.0.0). Subsequently, for each dataset, a simulation was run using as input the rearing conditions (temperature, trial duration, initial fish size, feed composition, ration size and feeding schedule) corresponding to the respective study. The resulting model predictions were plotted against the actual measurements and grouped based on the data type (weight, gaseous exchange, TAN excretion). In terms of assessment indicators, we computed two error measures for each type of data, the mean relative error (MRE) and the symmetric mean squared error (SMSE) given by

(9) $$\begin{array}{c} \text{MRE}=\frac{1}{n}\;\sum_{i=1}^{n}\frac{\left|{\hat{x}}_{i}-\;x_{i}\right|}{\left|x_{i}\right|} \end{array}$$

(10) $$\begin{array}{c} \text{SMSE}=\frac{1}{n}\;\sum_{i=1}^{n}\frac{{\left({\hat{x}}_{i}|-|\;|x_{i}\right)}^{2}}{{x_{i}}^{2}+{{\hat{x}}_{i}}^{2}}, \end{array}$$

where *x*_*i*_ is the observed value and ${\hat{x}}_{i}$ is the model prediction

## 3. Results

### 3.1. Fitting the Digestion Module

**Figure 2:**
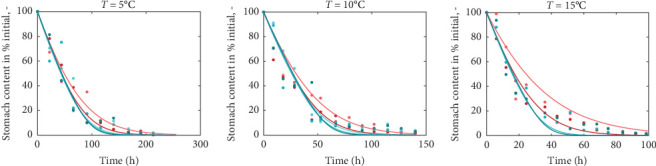
Stomach content in % of the initial amount as a function of time at three temperatures. Fish were fed ad libitum and *t* = 0 is the time feeding ceased. Points indicate observations and lines model predictions. Data on gastric evacuation obtained from From and Rasmussen [52]. The different colors indicate different fish sizes, with the light red to correspond to small fish and the dark teal to larger size.

**Figure 3:**
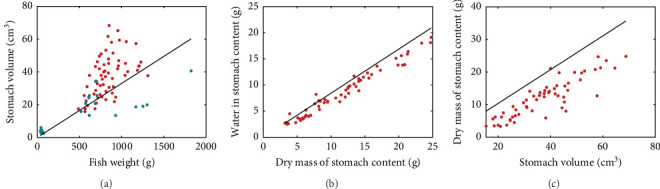
Relationship between stomach volume and fish weight (a), water in stomach content and dry mass of stomach content (b), and dry mass of stomach content and stomach volume (c). Points indicate observations and lines model predictions. Data from Pirhonen and Koskela [49] (red dots) and Ruohonen and Grove [53] (blue dots).

Using the rainbow trout (*Oncorhynchus mykiss*) as an example, we present the nutritional bioenergetics model performance. The DEB parameters of the abj-model without the digestion–assimilation module for *O*. *mykiss* are available online (AmP *Oncorhynchus mykiss* (v. 2017/10/30)) and are provided in the Supporting Information (Section S.1). The parameters linked to the digestion–assimilation module were estimated using literature data, while the core DEB parameters obtained online were fixed. All the parameter values and descriptions are provided in the Supporting Information (Section S.1). Model fits to those data are shown below including gastric evacuation duration (Figure 2), stomach volume for various fish weight (Figure 3a), water in stomach content that correspond to dry mass stomach content (Figure 3b) and stomach content dry mass and stomach volume (Figure 3c). Supporting Information Table S1 provides the values of the digestion–assimilation module parameters.

The data in Figure 2 correspond to gastric evacuation of fish ranging in size between 5 and 71 g at temperatures 5°C, 10°C, and 15°C. Gastric evacuation time decreases at higher temperatures. The model predictions reflect both the rate and the duration of gastric evacuation across the different temperatures and fish sizes. Larger fish empty the stomach faster compared to the smaller ones. This is also reflected in the data, but the pattern is not persistent.

Stomach volume shows a positive relationship with fish weight, although with considerable scatter (Figure 3a). Rainbow trout moisturises ingested dry food and Pirhonen and Koskela [49] showed that there is a linear relationship between water content of food and dry mass of stomach content (Figure 3b). The estimated slope is 0.8425, meaning that for each gram of ingested dry mass, 0.8425 g of water is added. The relationship between dry mass of stomach content and stomach volume is shown in Figure 3c. The model predicts the maximum storage capacity from the stomach volume.

**Figure 4:**
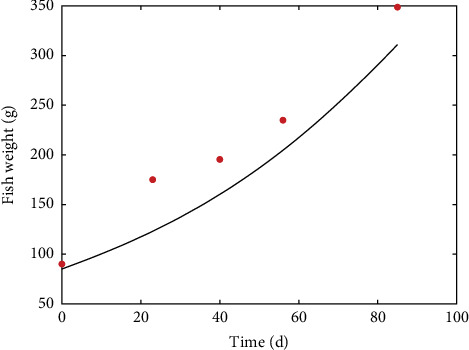
Weight increase of rainbow trout during a 85 day digestibility trial (points) compared to model predictions (line). Data from Zhu et al. [55].

Figure 4 shows the increase of weight over a period of 85 days in a digestibility trial when fish were fed twice a day 1.8% of body weight and raised at a mean temperature of 16°C. Model underestimation, especially at the beginning of the simulation, is likely due to temperature variations during the trial.

### 3.2. Model Validation

**Figure 5:**
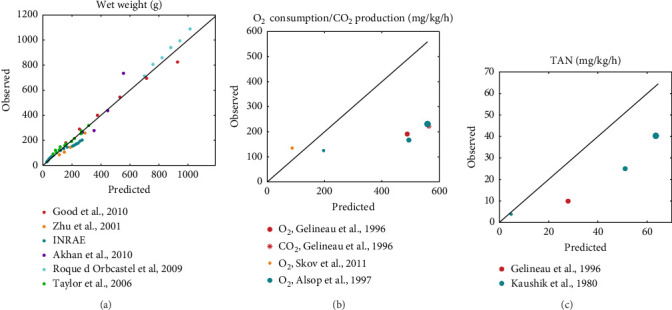
Comparison of DEB model predictions for trial observations of wet weight (a), O_2_ consumption and CO_2_ production (b) and total ammonia nitrogen (TAN) excretion (c) using as input trial rearing conditions (temperature, trial duration, initial fish size, feed composition, ration size and feeding schedule). Different colors indicate different literature datasets and varying marker sizes indicate different feeding conditions: satiation (large), restricted (medium) and starvation (small). The equality line serves as a reference, denoting perfect agreement between observed and predicted values.

Literature datasets on weight, oxygen consumption, carbon dioxide production and TAN excretion were used for model validation. These datasets were different from the ones used for the estimation of model parameters in order to allow for an independent evaluation of model performance. They included various fish sizes, temperatures and diets that differed in both quantity and quality, which were used as input to perform simulations using the estimated rainbow trout parameters. The resulted predictions are plotted against the actual observations while the equality line (*y* = *x*) is also inserted to visually represent perfect agreement between model predictions and observations (Figure 5). Points scattered around this line reflect the extent of deviations between predictions and observations.

On average, the model predicted the body weight (MRE = 0.019, SMSE = 0.016) than gaseous exchange (MRE = 0.86, SMSE = 0.198) and nitrogenous waste (MRE = 0.923, SMSE = 0.176). Particularly for growth (Figure 5a), the points were scattered around the equality line, indicating no particular bias for over- or under-estimation. However, the model tended to overestimate oxygen consumption, particularly in the cases involving feeding close to satiation (large marker size). This bias was not exhibited at low feeding levels. A similar trend as that observed for oxygen consumption was noted for the TAN excretion but with less deviation from the observed values. Overall, the model performed well and was able to capture the diverse nature of the inputs of the validation datasets.

### 3.3. Model Outputs

**Figure 6:**
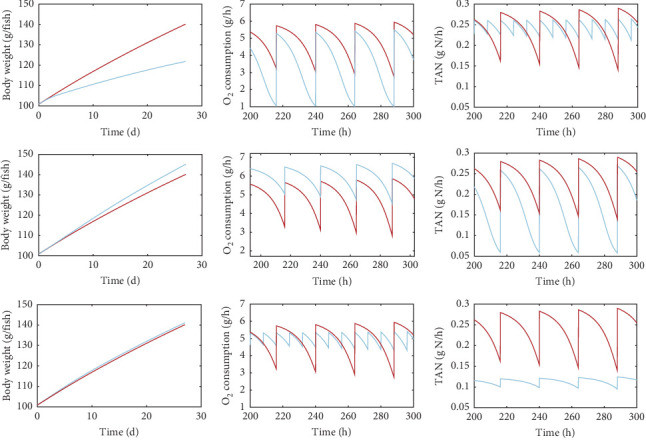
Simulations of fish weight, O_2_ consumption, and total ammonia nitrogen (TAN) production over a 30-day experiment under different feeding conditions. Effects of feeding level (top row): 1.2% (red) and 0.8% (blue) of body weight per day, feed composition is 45% protein, 22% fat, 19% carbohydrates and feeding frequency once per day. Effects of feed composition (middle row): 45% protein, 22% fat, 19% carbohydrates (red) and 30% protein, 38% fat, 19% carbohydrates (blue), for a feeding level of 1.2% of bodyweight and feeding frequency once per day. Effects of feeding frequency (bottom row): once (red) and three times per day (blue), (feeding level 1.2% of body weight and feed composition is 45% protein, 22% fat and 19% carbohydrates. Simulations are performed at *T* = 15°C and for a group of fish *N* = 100.

In this section, we present examples of model simulations to demonstrate the capabilities of the model.  Figure 6 illustrates some of the main model outputs for a specific set of inputs, thus, representing scenarios that end-users could simulate using the model. In addition to the evolution of fish weight under different feeding levels, feed compositions and feeding schedules during the simulation period, the model allows simulations of oxygen consumption and waste production. The resolution of the output could be hourly or daily; the hourly output will show fluctuations (diurnal variation) in waste production due to the applied feeding schedule. Examples of how the feeding level, the feed composition and the feeding frequency would affect fish growth, oxygen consumption and TAN production are shown in Figure 6. It is also important to note that the model can simulate without additional assumptions other quantities of interest for aquaculture, not shown here. Such outputs include variables like the undigested solid wastes, CO_2_ production and food conversion ratio.

#### 3.3.1. Growth

As expected, the modelled growth (Figure 6, left column) is strongly linked to the feeding level (expressed as % body weight per day), with a higher ratio resulting in higher weight gain. Diet composition also affects fish weight, with the diet moderately richer in fat, and thus richer in energy, resulting in increased growth. On the contrary, growth does not seem to be affected considerably by the feeding frequency.

#### 3.3.2. Oxygen Consumption

With respect to the simulated oxygen consumption (Figure 6, middle column), it is positively correlated with the feeding level, with the higher ration scenario resulting in notably higher oxygen demands. Similarly, diet composition appears to play a significant role, with substantial shifts in oxygen consumption associated with the different diets. Interestingly, the most prominent effect is that of feeding frequency. Feeding frequency tends to determine the magnitude of fluctuations in oxygen demand during the day with the lower feeding frequency resulting in smoother variations compared to the higher frequency schedule.

#### 3.3.3. TAN Production

Regarding waste (Figure 6, right column), the production of TAN is directly related to both feeding level and diet composition. Specifically, increasing feeding levels correlate positively with TAN production. In addition, diet composition has a significant impact on the production of nitrogenous waste, with a diet richer in protein leading to higher TAN production. Finally, similarly to the oxygen production, the effect of feeding frequency is particularly pronounced for TAN production since an increase in feeding frequency (three meals per day instead of one) here resulted in lower daily fluctuations of gas exchanges and waste production.

However, as will be reported in the protein-to-energy ratio section, growth, oxygen demand and waste production are heavily affected by the dietary protein-to-fat ratio intake, which determines food assimilation and, therefore, the energy that remains available for growth.

#### 3.3.4. Digestible Protein to Digestible Energy (DP/DE) Ratio

**Figure 7:**
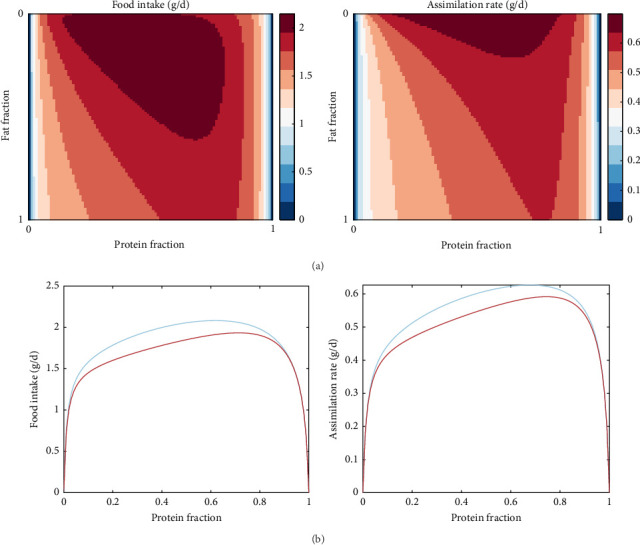
Effects of diet composition on food intake and assimilation rates. (a) Heatmaps for a range of fractions of protein in the food and fractions of fat in the non-protein component of food. (b) Food intake and assimilation rates (expressed in ash-free dry mass, g/d) as a function of the fraction of protein in the food at two constant fractions of fat and carbohydrate contents for the non-protein component of the food (fractions of 0.7 fat (red) and 0.3 fat (blue) for the remaining food once protein is accounted for). Simulations were done for 150 g trout, at 14°C and for ADC of protein, fat and carbohydrates being 0.9, 0.9 and 0.7, respectively.

One of the model's emerging properties is its ability to capture the effects of food composition on food intake and assimilation, which essentially translates to the effects of digestible protein-to-digestible energy (DP:DE) ratio in the diet. Specifically, Figure 7a shows the food intake and assimilation rate (dry mass, g/d) for gradients of protein as well as the fraction of fat on the non-protein diet component. While the patterns are similar, assimilation follows feed intake with slight differences and lower numerical values, which should be expected given the overhead costs and the overall conversion efficiency of the process. Notably, assimilation is nullified for diets extremely low or high in protein, while for diets of intermediate protein levels, the defining factor is the energy content (the fat to carbohydrate ratio). In fact, diets extremely high in fat seem also to result in poor intake and assimilation rates; however, this can be mitigated to a certain extent by selecting appropriate protein levels. To better illustrate this model property, Figure 7b shows an example of a high- and a low-fat diet for the non-protein component of the food. The shape of the food intake and assimilation rates as functions of the protein fraction in the food resembles inverted parabola; food either low or high in protein results in low assimilation rates. Moreover, the shapes of the curve are influenced by the non-protein component of the food, meaning that for a fixed fraction of protein, the rates depend on the fat and carbohydrate contents (which determine energy content). It therefore follows that for a given ratio of fats and carbohydrates, there exists a specific protein fraction that maximises assimilation and food intake.

**Figure 8:**
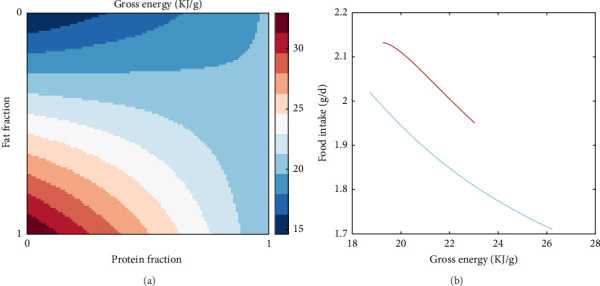
(a) Simulated effects of nutrient composition (protein, fat and carbohydrates) on the gross energy content of the diet for a range of protein fractions in the food and fat fractions in the non-protein component (as calculated from eq. A7). (b) Food intake (expressed in ash-free dry mass, g/d) as function of gross energy at two protein fraction levels: 60% (red) and 30% (blue) and fat fractions in the non-protein component in the range of 20%–80%.

A direct consequence of the differences in the combustible energy contents of proteins, fats and carbohydrates is that the gross energy content of the food is heavily dependent on food composition, as described in Equation A7. This is visualised in Figure 8a where the gross energy content of the food increases with increasing fat in the non-protein component in the food (i.e., decreasing carbohydrates) while it shows little variation for extremely protein-rich diets. An emerging property of the presented model is that it captures the effects of the energetic content of food on food intake (Figure 8b). Specifically, food intake and dietary energy content are inversely related with diets low in energy requiring higher feeding levels to achieve satiation.

## 4. Discussion

The use of models for fish metabolism is increasingly encouraged in aquaculture, both from a production (precision farming) and ethical perspective (3Rs) [23, 63]. In this article, we have presented a model describing the nutritional bioenergetics of fish in aquaculture with the intention of providing a useful in silico tool for farm operators and fish researchers. The model is based on DEB theory, which provides a mechanistic framework to describe individual metabolism [32]. The model explicitly describes the flow of energy and chemical elements in the organism and its utilisation in various processes such as ingestion, assimilation, maintenance and growth. As such, it can predict changes in measurable variables of interest for aquaculture such as feeding, respiration, weight production and weight gain under different temperatures and various feeding conditions (feeding regimes), both in terms of quantity and macronutrient composition.

As seen from the use case examples provided in the previous sections, the model has substantial capabilities in predicting variables of interest under diverse conditions and feeding regimes. Some of them such as weight or biomass gain are of universal interest for producers and therefore model predictions could provide useful insights into the effects of temperature or feeding schedule [39, 64]. Other variables such as waste production may be highly relevant for specific production systems such as recirculating aquaculture systems where the fate of nitrogenous waste must be closely monitored [65], and thus they may offer means of managing populations in tanks or means of evaluating system capacity prior to conducting fish trials. In addition, oxygen consumption is a vital parameter for fish husbandry and welfare irrespective of production [66, 67]. By providing predictions of oxygen requirements, the tool could be used to identify appropriate population numbers for a given experimental or production setup under different feeding levels, composition or temperatures. Finally, the model's feature to predict waste production, including the aforementioned TAN but also carbon dioxide and solid wastes, may extend the application of the model to other fields of research such as environmental ecology. For instance, the nutrient dynamics in the water column and potential effects on the marine communities surrounding farm sites could be investigated while the model could aid in the determination of the ecological carrying capacity for the designation of new farming sites [68].

As demonstrated in the model validation section, the model is accurate in its predictions of growth especially considering the diverse nature of the datasets used for this process (MRE of 0.019). These datasets were obtained from published studies across different production systems and varying experimental protocols, including both controlled experimental conditions and commercial farms. While, admittedly, predictions such as those for gaseous exchange and TAN production are less accurate (MRE of 0.86 and 0.92), there was generally agreements between model predictions and the observation used for validation with no systematic bias exhibited in either of the considered variables. This increases the robustness of the model and the confidence in its future use, which has been recognised as an important element in tool development and its potential for adoption and acceptance by the target users [69]. The highest deviations between model predictions and observation data from experiments were observed for smaller-sized fish and for ad libitum feeding conditions. Unsurprisingly, most of the datasets used for estimation of the core DEB and digestion model parameters pertained to large fish under well-defined feeding conditions. Therefore, deviations by model predictions can likely be partially explained by methodological fuzziness in the definition of ad libitum feeding across different studies, as well as concomitant insufficiencies in the available data regarding the digestion process, particularly, the maximum stomach capacity. Furthermore, the diverse nature of protocols used in fish research can cause substantial scatter in the studied variables, which affects the accuracy of the estimated parameters. For instance, the moisturising coefficient used here was calculated from the study of Pirhonen and Koskela [49]. Yet, in a recent study, the same coefficient was found ~10% lower in trout stomach while, interestingly, the value for the proximal intestine was comparable to the one used here [50]. Moreover, it has been shown that water balance can be notably affected by the dietary electrolyte balance and particularly the DE:DP ratio [70]. This demonstrates that there are further nuances in the digestion process, which however exceed the scope of our work. However, it is evident that in order to improve parameter estimates and prediction accuracy for the case study given here, as well as for the application of the proposed nutritional model to other species, it is important to enrich the nutritional bioenergetic model with early life stage data as well as conduct dedicated experiments to increase clarity with respect to the digestion parameters. In this direction, the employment of new technologies such machine learning, use of genomic supported devices and exploitation of big data may also contribute to the provision of datasets for model development, especially in areas where direct experimentation and observation are not feasible.

One critical consideration in fish nutrition and feed formulation is the energy-to-protein ratio. The optimal ratio is highly species-specific and needs to be experimentally determined for each species [71–73]. Feeds with excessive energy-to-protein content and vice versa are known to result in reduced weight gain because the fish may eat as much as they can, but depending on the case, either the protein or the energy they can retain is not sufficient to cover their growth requirements [74, 75]. Moreover, the source of energy, whether it is predominantly derived from fats, protein or carbohydrates further influences its utilisation by the fish. In fact, it is known that diets with a balanced combination of the above macronutrients and, therefore, balanced elemental composition offer advantages in terms of digestion and metabolism as well as immunology and oxidative capacity compared to diets heavily skewed towards one or the other [76–78]. Interestingly, these two important aspects of fish nutrition are captured by the model presented here. Although the intent was not to explicitly model the effect of different macronutrients on assimilation, this was observed as an emerging property (Figure 7). Specifically, the relation between assimilation rate and protein to energy ratio formed an inverted parabola pointing to an optimum ratio with detrimental effects for diets excessively low or high in protein content, as is also known from literature [79]. Moreover, this point was dependent upon the relative content of lipids and carbohydrates. In addition, the model was able to capture the relationship between the food intake and the energy content of the food, suggesting lower feed intake for high energy diets; a pattern that has been documented experimentally for trout [80] and other fish [81]. Considering the current trends in fish nutrition for the reduction of protein or replacement with alternative to fishmeal sources as well as the increasing of carbohydrate inclusion to replace fish oil [77, 82–84], this model could act as an initial screening tool for identifying sensible ranges for energy to protein ratios and acceptable combinations of lipids and carbohydrates in experimental feeds. In turn, this could contribute to optimising experimental design in nutritional studies, resulting in effective utilisation of resources and reduction in the number of animals used according the 3R guidelines.

A novelty of the present model is the waste outputs, namely the nitrogenous waste excreted via the gills or in the faeces, as well as the solids and the carbon dioxide production (not shown here) which stem from a careful accounting of the mass balances of the elements C, H, O and N at the molecular level provided by the DEB framework. Furthermore, these outputs may be provided at resolutions lower than a day, thus, accounting for different feeding schedules and allowing a close monitoring of these compounds throughout the day. This is a substantial addition to existing bioenergetic models for fish. While more generic bioenergetic approaches may be suitable for certain aquaculture settings and offer substantial accuracy in growth and feeding predictions [14, 27, 28], it is the mechanistic nature of the detailed nutritional model that allows the description of more complex processes. The present approach strived to achieve a balance between realism and complexity, simplifying the gut and nutrient dynamics to a degree that allowed satisfactory parametrisation with the available data while retaining functionality. More detailed extensions, that exceed the scope of this work but would be worth exploring in the future, include modelling the effects of the quality of macronutrients (amino-acid and lipid profiles) on the metabolic processes, the explicit modelling of the dietary ash content, as well as the effects of nutrition on the body composition of the fish, as it has been pointed elsewhere [28]. While data availability and some of the nuances of the digestion process that require further attention have been discussed, it is important to highlight some additional model limitations to avoid misuse of the model. Specifically, the assumptions made for the derivation of the digestion module such as the consolidation of the alimentary track as 'gut' the homogeneity of gut content which excludes effects of particle size and/or composition on digestion rate, the exclusion of ash in reserve dynamics, the inclusion of a water intake correction during ingestion and the dependencies of maximum stomach capacity on stomach geometry and food composition need to be kept in mind during the use of the model and evaluated appropriately when further model applications are considered. Furthermore, it is crucial for users to evaluate the model performance according to the required level of accuracy for each potential application. For instance, while fitting stomach volume – dry stomach content data (Figure 3), there was a systematic model offset with our model overestimating the physical capacity of the stomach. This could be due to different allometric relations applying at younger fish stages. In the absence of data for the early life stage, we refrain from making further assumptions on the relationship between volume and content. However, this issue, which is worth exploring in the future, may cause some deviations in other model predictions. Namely, model validation showed that while growth was predicted accurately, TAN and even more so O_2_ and CO_2_ where overestimated. This documentation allows for placing different levels of confidence for the various types of the model predictions, meaning that while they can all be used for initial screening of scenarios, their application may be inappropriate in certain settings. For instance, good estimates of O_2_/CO_2_ are needed to evaluate the contribution of macronutrients on fish metabolism, and therefore, if one uses the model for precise nutritional work, it is important to take into account the associated overestimation and/or uncertainty in the model predictions.

Due to its generality, the mechanistic framework of DEB theory allows easy application of the developed models to other species while its modular design facilitates its integration with other models. At present, we have used the example of rainbow trout to showcase the design and capabilities of the model. Furthermore, we have applied the model to other species, such as Atlantic salmon and gilthead seabream, obtaining similar results (AquaExcel2020 deliverable) [85]; additional species are being considered as well. Irrespective of its use as a standalone model, the DEB model with the add-on module for digestion has been implemented as a constituent model in “Virtual Laboratory”, a platform dedicated to supporting actual and virtual research experiments in aquaculture (https://aquaexcel2020.eu/virtual-laboratory). The tool, which was originally developed as part of the AquaExcel2020 project and is currently under further development (AquaExcel3.0), allows the simulation of experiments with fish by integrating a set of numerical models such as metabolic, behavioural, water treatment and water flow models.

## 5. Conclusions

This work aimed at providing an in silico tool for fish researchers and aquaculture farm operators by modelling the nutritional bioenergetics of fish. The proposed model relies on a mechanistic framework (DEB theory) that explicitly describes the flow of energy and chemical elements in the organism and its utilisation in various processes such as ingestion, assimilation, maintenance and growth. A novelty of the current work is the development a digestion module which adds functionality by allowing the description of nutritional effects on metabolism, and specifically linking feeding level, feeding frequency and feed composition to variables of interest for aquaculture including growth, oxygen consumption, carbon dioxide, ammonia and solid waste production, under various rearing conditions. The presented approach is an extension of the typical DEB models in that it includes a digestion module where the protein and non-protein food components contribute to assimilation via the concept of a SU. Showcasing model capabilities using the rainbow trout as an example demonstrates that the model captures the observed patterns in these variables under diverse validation scenarios particularly well for growth (MRE of 0.019) and to a lesser extent for nitrogenous waste and gaseous exchange (MRE of 0.86 and 0.92 respectively). The model can also, without further assumptions, quantify the effects of the dietary protein-to-energy ratio to food intake and assimilation as an emergent model property, namely it predicts decrease of food intake for high energy diets. While shortcomings, improvements and possible extensions of the model are discussed, overall, the presented approach managed to achieve a balance between realism and complexity, simplifying the gut and nutrient dynamics to a degree that allowed satisfactory parametrisation with the available data while retaining functionality. Potential applications of the model include simulation of growth scenarios under diverse rearing conditions, identification of sensible ranges for energy to protein ratios and acceptable combinations of lipids and carbohydrates in experimental feeds, monitoring and management of waste and oxygen in closed systems, assessment of environmental effects and generally, the testing of a diverse range of scenarios under a virtual laboratory setting. The mechanistic nature of the model offers generality, thus facilitating its application to other species while allowing for the description of complex processes, which constitutes an important contribution to existing bioenergetic models for fish.

## Data Availability

The data that support the findings of this study are available upon request from the corresponding author. The data are not publicly available due to privacy or ethical restrictions.

## Supporting Information

Supporting Information The manuscript is accompanied by a supporting information file containing additional methodological details on model equations and derivations and is divided into three sections. The first section (S.1) summarises the model equations (state variables, the energy fluxes and the dynamics of an individual fish), lists the model parameters and provides the parameter values. The second section (S.2) describes the mathematical derivation of the assimilation rate, the third (S.3) the derivation of chemical indices for organic compounds and the fourth (S.4) summarises the methodological details for the datasets used in model validation.
