# Supplementary material for: A Nutritional Bioenergetic Model for Farmed Fish: Effects of Food Composition on Growth, Oxygen Consumption and Waste Production
Source: Aquac Nutr. 2025 Jul 26;2025:9010939. doi: 10.1155/anu/9010939 (PMC12317818; doi:10.1155/anu/9010939)
Supplement: Supporting Information — The manuscript is accompanied by a supporting information file containing additional methodological details on model equations and derivations and is divided into three sections. The first section (S.1) summarises the model equations (state variables, the energy fluxes and the dynamics of an individual fish), lists the model parameters and provides the parameter values. The second section (S.2) describes the mathematical derivation of the assimilation rate, the third (S.3) the derivation of chemical indices for organic compounds and the fourth (S.4) summarises the methodological details for the datasets used in model validation. [file 9010939.f1.pdf]

**A nutritional bioenergetic model for farmed fish: Effects of food composition on growth, oxygen consumption and waste production**

**Supplementary Material**

Orestis Stavrakidis-Zachou[1], Ep H. Eding[2], Nikos Papandroulakis[1], Konstadia Lika[3]

[1] Institute of Marine Biology, Biotechnology and Aquaculture, Hellenic Centre for Marine Research, Heraklion, Greece

[2]Aquaculture and Fisheries Group, Wageningen University & Research, Wageningen, The Netherlands

[3] Department of Biology, University of Crete, Heraklion, Greece

Journal: Aquaculture Nutrition
Corresponding author email: ostavrak@hcmr.gr

*S.1. Model equations and parameters*

This section summarizes in Table S1 the model equations and presents in Table S3 all model parameters. For a more comprehensive description of the DEB theory and a full list of the equations and the nomenclature used we refer to Kooijman (Kooijman, 2010) and Stavrakidis-Zachou (Stavrakidis-Zachou et al., 2019). Most species with a larval phase which show metabolic acceleration at, or soon after, birth for a period which frequently coincides with completeness of morphological metamorphosis are modelled using the a-models (Marques et al., 2018). Fish species which show metabolic acceleration between birth (start of exogenous feeding) and metamorphosis are modelled with the abj-model. Metamorphosis is before puberty and occurs at maturity level $E_H^j$. According to abj-model assumptions, between birth and metamorphosis the individual follows the rules for V1-morphy, while before and after acceleration growth is isomorphic.

The V1-morphic mode affects the relationship between surface area and structural volume; namely, the surface area is proportional to structural volume, while in isomorphic mode surface area is proportional to structural volume to the power 2/3. Changes in shape affect the surface-specific maximum acceleration rate, the surface-specific maximum digestion rate, and the energy conductance via the acceleration factor $s_M$. The fact that only these parameters are affected follows from the structure of DEB theory. The acceleration factor, $s_M$, equals one for embryos and pre-larvae stage, $L/L_b$ during acceleration, and $L_j/L_b$ after acceleration. At the end of this period, the aforementioned parameters remain constant again (apart from effects of temperature) but differ from the original value by the acceleration factor $s_M$, which depends on food availability during the acceleration period. Consequently, the dynamics (Table A1) will change via the fluxes $\dot{p}_A$ and $\dot{p}_C$.

All physiological rates depend on temperature. For a species-specific range of temperatures, the temperature effect is quantified by the Arrhenius relationship (Kooijman, 2010). For $T_A$ the species-specific Arrhenius temperature, the rate of a physiological process $\dot{k}$ at temperature T is given by

$$\dot{k}(T) = \dot{k}_1 \exp\left(\frac{T_A}{T_1} - \frac{T_A}{T}\right)$$

where $\dot{k}_1$ is the rate at a chosen reference temperature, here $T_1$=293K. The exponential term is the correction term around the reference temperature and acts at temperatures within the tolerance temperature range for the species.

Table S1: State variables, energy fluxes and dynamics of the DEB model. Brackets [.] indicate quantities expressed per unit of structural volume and braces {.} per unit of structural surface area.

| | |
|---|---|
| *State variables* | |
| $V$, $L = V^{1/3}$ | Structural body volume ($cm^3$), Volumetric structural length (cm) |
| $E$, $[E] = E / V$ | Energy in reserve (J), Reserve density ($J/cm^3$) |
| $E_H$, $E_R$ | Energy investment (J) into maturation, - to reproduction |
| $M_X$ | Mass content of the food in the stomach (mol) |
| *Fluxes* | |
| $\dot{p}_A$ | Assimilation rate: $\dot{p}_A = \{\dot{p}_{Am}^d\} f_X L^2$, with $f_X = \frac{M_X}{M_X + M_K^X}$ and $M_K^X = \frac{\{\dot{J}_{EAm}^d\}}{\{\dot{J}_{Xgm}\}} \left( (y_{EX_P} a_P)^{-1} + (y_{EX_{nP}}(1 - a_P))^{-1} - (y_{EX_P} a_P + y_{EX_{nP}}(1 - a_P))^{-1} \right)$ |
| $\dot{p}_C$ | Reserve mobilization rate: $L^3 [E](\dot{v}/L - \dot{r})$ with $\dot{r} = \frac{\frac{\kappa [E] \dot{v}}{L} - \dot{p}_S}{[E_G] + [E]\kappa}$ |
| $\dot{p}_S$ | Somatic maintenance rate: $[\dot{p}_M] L^3$ |
| $\dot{p}_J$ | Maturity maintenance rate: $\dot{k}_J \min\{E_H, E_H^p\}$ |
| $\dot{p}_G$ | Growth rate: $\kappa \dot{p}_C - \dot{p}_S$ |
| $\dot{p}_R$ | Energy flux to maturation/reproduction: $(1 - \kappa)\dot{p}_C - \dot{p}_J$ |
| $\dot{p}_D$ | Dissipating power: $\dot{p}_S + \dot{p}_J + \dot{p}_R$ (larvae/juveniles); $\dot{p}_S + \dot{p}_J + (1 - \kappa_R)\dot{p}_R$ (adults) |
| *Dynamics* | |
| $\frac{d}{dt} V = \dot{r} V$ | |
| $\frac{d}{dt}[E] = [\dot{p}_A] - [E]\dot{v}/L$ | |
| $\frac{d}{dt} E_H = \dot{p}_R (E_H < E_H^p)$ | |
| $\frac{d}{dt} E_R = \dot{p}_R (E_H \geq E_H^p)$ | |
| $\frac{d}{dt} M_X = -(y_{X_P E} + y_{X_{nP} E} + y_{PE}) \dot{p}_A / \mu_E$ | |

Table S2: Description of model parameters. The values are given for rainbow trout (*Oncorhynchus mykiss*) at 20°C.

| Symbol | Value | Units | Description |
|---|---|---|---|
| | | | |
| **DEB parameters[1]** | | | |
| $\dot{v}$ | 0.03 | cm/d | Energy conductance |
| $\kappa$ | 0.62 | - | Allocation fraction to soma |
| $\kappa_R$ | 0.95 | - | Reproduction efficiency |
| $[\dot{p}_M]$ | 344 | J/cm³.d | Volume-specific somatic maintenance rate |
| $[E_G]$ | 5268 | J/cm³ | Specific costs for structure |
| $E_H^b, E_H^j, E_H^p$ | 43, 854, 388·10⁴ | J | Maturity threshold at birth, metamorphosis, puberty[4] |
| $\dot{k}_J$ | 0.002 | 1/d | Maturity maintenance rate coefficient |
| $\mu_*$ | | J/mol | Chemical potentials of $*$ = X(food), P(product), V(structure), E(reserves) |
| $w_*$ | | g/mol | molecular weights of $*$ |
| $d_*$ | | g/cm³ | specific density of $*$ |
| $n_{C*}, n_{H*}, n_{O*}, n_{N*}$ | | - | chemical index of elements (C,H,O,N) in organic compounds * |
| $T_A$ | 8000 | K | Arrhenius temperature |
| | | | |
| **Digestion parameters[2]** | | | |
| $\delta_g$ | 0.588 | - | Stomach-volume shape coefficient |
| $\{\dot{j}_{EAm}^d\}$ | 4.291 ·10⁻³ | mol/cm² .d | Surface-area-specific max assimilation rate |
| $\{\dot{J}_{Xgm}\}$ | 1.828 | 1/cm².d | Surface-area-specific max digestion rate |
| $y_{HX_d}$ | 0.8425 | - | Food moisturizing coefficient: grams of water required in the stomach to moisturize the ingested food |
| $\{\dot{p}_{Am}^d\} = \mu_E\{\dot{j}_{EA_m}^d\}$ | | J/cm² d | Surface-area-specific max assimilation rate |
| $[M_{gm}] = \left(y_{HX_d}\frac{d_{Xd}}{d_H}+1\right)^{-1}\frac{d_{Xd}}{w_X}\delta_g$ | | mol/cm³ | Volume-specific max food capacity of the stomach (dry weight) |
| | | | |
| **Food specific parameters[3]** | | | |
| $\kappa_{X_P}, \kappa_{X_{nP}}, \kappa_{X_{NFE}}$ | | - | Fraction of food protein, lipid and carbohydrates fixed in reserve |
| $\kappa_X$ | | - | Fraction of food energy fixed in reserve |
| $\kappa_P$ | | - | Faecation efficiency of food to faeces |
| $\mu_*$ | | J/mol | Chemical potentials of $*$ = X(food), P(faeces) |
| $w_*$ | | g/mol | molecular weights of $*$ |
| $d_*$ | | g/cm³ | specific density of $*$ |
| $n_{C*}, n_{H*}, n_{O*}, n_{N*}$ | | - | chemical index of elements (C,H,O,N) in organic compounds * |
| $a_P$ | | - | Fraction of protein in food |
| $\kappa_{X_{nP}} = \kappa_{X_L}a_L + \kappa_{X_{NFE}}(1-a_L)$ | | - | Digestion efficiency of non-protein to reserves, with $a_L$ the fraction of lipids in the non-protein part of food |
| $y_{EX_P} = \kappa_{X_P}w_{X_P}/w_E$ | | mol P/mol E | Yield of reserve on assimilated protein |
| $y_{EX_{nP}} = \kappa_{X_{nP}}w_{X_{nP}}/w_E$ | | mol nP/mol E | Yield of reserve on assimilated non-protein |
| $\kappa_X = \theta_P\kappa_{X_P} + \theta_L\kappa_{X_L} + \theta_A\kappa_{X_A} + \theta_{NFE}\kappa_{X_{NFE}}$ | | - | Digestion efficiency of food to reserves, where θ are the fractions of protein, lipid, |

| | | | |
|---|---|---|---|
| | | | ash, and carbohydrates in food and κ their respective digestibilities. |
| $\kappa_P = \theta_P(1-\kappa_{X_P}) + \theta_L(1-\kappa_{X_L}) + \theta_A(1-\kappa_{X_A}) + \theta_{NFE}(1-\kappa_{X_{NFE}})$ | | - | Digestion efficiency of food to faeces, where θ are the fractions of protein, lipid, ash, and carbohydrates in food and $\kappa$ their respective digestibilities. |
| $y_{PE} = \frac{\kappa_P \mu_E}{\kappa_X \mu_P}$ | | - | Yield of faeces on reserve |
| $k_X$ | | - | (wet) Weight of food as fraction of body (wet) weight |

[1] parameter values were retrieved from AmP *Oncorhynchus mykiss*, version 30 Oct 2017 (bio.vu.nl/thb/deb/deblab/add_my_pet/entries_web/Oncorhynchus_mykiss/Oncorhynchus_mykiss_res.html).
[2] parameter values were estimated in this study
[3] values are food specific and are derived based on formulae given in appendix S3.
[4] sex-based differences in parameter values were not considered.

## *S.2. Derivation of the assimilation rate*

The shape of the digestive system resembles that of a (tube) cylinder. Thus, the volume of the alimentary track of length $L_\lambda$ and diameter $L_\varphi$ is $V_g = \pi L_\lambda L_\varphi^2/4$ and the surface area of contact between the stomach and its content is $A_g = \pi L_\lambda L_\varphi$. Following Kooijman's (Kooijman, 2010) approach, we assume that the secretion rate of enzymes is constant, and the deactivation kinetics is a first-order process. The dynamics of the amount of active enzymes, $M_g$, in the stomach, is then given by $\frac{d}{dt}M_g = \{\dot{J}_g\}\,\pi L_\lambda L_\varphi - \dot{k}_g M_g$, where $\{\dot{J}_g\}$ is the constant secretion rate of enzyme per unit of stomach wall surface area and $\dot{k}_g$ is the decay rate of enzyme activity. If we assume that the concentration of active enzymes reaches a steady state fast, the amount of active enzymes at steady state is $M_g = \{\dot{J}_g\}\,\pi L_\lambda L_\varphi/\dot{k}_g$. For isomorphs, we can assume that the length $L_\lambda$ and diameter $L_\varphi$ are proportional to structural length $L = V^{1/3}$. Consequently, the amount of active enzymes at steady state is proportional to surface area, $M_g \propto L^2$. Enzymes catalyse the breakdown of food, and produce products that will then be absorbed through the digestive wall and form the generalized reserve molecules. Let $M_X$ be the mass of food (in mol) in the stomach. The rate at which the products are produced by the digestion process is proportional to $M_g M_X$, i.e., $\dot{J}_d = \{\dot{J}_{Xgm}\}L^2 M_X$, where $\{\dot{J}_{Xgm}\}$ is the maximum surface-specific rate of digestion.

Food is a mixture of organic (proteins, lipids, and carbohydrates) and inorganic (ash) components. For simplicity, the present model assumes that only the organic compounds partake in the formation of reserves and therefore the ash content is excluded from reserve dynamics. However, based on its apparent digestibility coefficient and the calculation of the digestion efficiency of food to faeces, ash content can be traced in the solid waste production. For the mixture of organics, suppose that a fraction $a_P$ of food is protein and the remaining $1-a_P$ are the lipids and carbohydrates. From now on we will call them protein, $X_P$, and non-protein, $X_{nP}$, component. Consequently, a fraction $a_P$ of the food in the stomach is protein and the remaining $1-a_P$ non-protein. The composition of each compound is represented as a "generalized" compound with fixed stoichiometry. The protein compound of the food is denoted by $CH_{n_{HX_P}}O_{n_{OX_P}}N_{n_{NX_P}}$, the

non-protein by $CH_{n_{HX_{nP}}}O_{n_{OX_{nP}}}$, the reserves by $CH_{n_{HE}}O_{n_{OE}}N_{n_{NE}}$ and the faeces by $CH_{n_{HP}}O_{n_{OP}}N_{n_{NP}}$. The assimilation process can be described by the macro-chemical equation:

$$y_{EX_P}\, CH_{n_{HX_P}}O_{n_{OX_P}}N_{n_{NX_P}} + y_{EX_{nP}}\, CH_{n_{HX_{nP}}}O_{n_{OX_{nP}}} \rightarrow \\ CH_{n_{HE}}O_{n_{OE}}N_{n_{NE}} + y_{PE}CH_{n_{HP}}O_{n_{OP}}N_{n_{NP}} + \text{mineral products } (\mathrm{H_2O},\ \mathrm{CO_2}, \mathrm{N} - \text{wastes})$$

where $y_{EX_P}$ and $y_{EX_{nP}}$ are, respectively, the molecules of protein and non-protein required to form a molecule of reserve and $y_{PE}$ the molecules of feaces produced.

The absorption of the products through the alimentary track and transformation into reserves (assimilation process) is modelled using the synthesizing unit (SU) concept of DEB theory (Kooijman, 2010). A SU can be in the unbound state, waiting for the arrival of one or more substrates, or in the bound state, processing those substrates. We assume that the two complementary substrates of protein and non-protein are processed in parallel to produce the generalized reserves, $E$. A SU processing two substrates can be either in binding or processing state. Let $\theta_{..}$ be the fraction of SUs in the binding state, waiting for the required molecules of protein or non-protein to be bound, $\theta_{P.}$ and $\theta_{.nP}$ the fraction of SUs waiting for molecules of the missing substrate to be bound, and $\theta_{PnP}$ the fraction of SUs in the processing state.

Digestive enzymes break down food particles, $M_X$, which are then bound to free SUs to form the reserves. Let $j_{X_P}$and $j_{X_{nP}}$ be the arrival fluxes of the protein and non-protein substrates. The interactions of the two substrates into products (reserves and faeces) and the dynamics of the SUs are given in Figure S1. Assuming rapid convergence to steady state, the production flux then amounts to $\dot{J}_{EA} = \dot{k}\theta^*_{PnP}$, where $\theta^*_{PnP}$ is the fraction of SUs in the processing state.
Thus,

$$\dot{J}_{EA} = \frac{1}{\dot{k}^{-1} + \left(y_{EX_P} j_{X_P}\right)^{-1} + \left(y_{EX_{nP}} j_{X_{nP}}\right)^{-1} - \left(y_{EX_P} j_{X_P} + y_{EX_{nP}} j_{X_{nP}}\right)^{-1}} \qquad \text{(S1)}$$

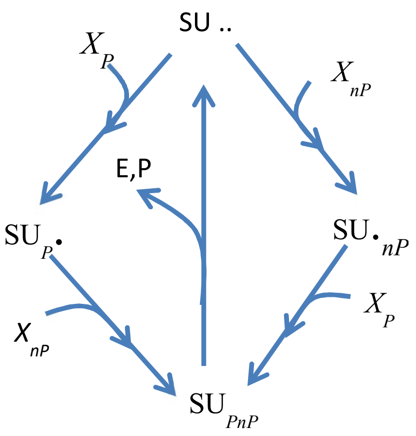


Dynamics of the fractions of the SUs at different states.

$$\frac{d\theta_{..}}{dt} = -(y_{EX_P}j_{X_P} + y_{EX_{nP}}j_{X_{nP}})\theta_{..} + \dot{k}\theta_{PnP}$$
$$\frac{d\theta_{P.}}{dt} = y_{EX_P}j_{X_P}\theta_{..} - y_{EX_{nP}}j_{X_{nP}}\theta_{P.}$$
$$\frac{d\theta_{.nP}}{dt} = y_{EX_{nP}}j_{X_{nP}}\theta_{..} - y_{EX_P}j_{X_P}\theta_{.nP}$$
$$\frac{d\theta_{PnP}}{dt} = y_{EX_{nP}}j_{X_{nP}}\theta_{P.} + y_{EX_P}j_{X_P}\theta_{.nP} - \dot{k}\theta_{PnP}$$
$$\theta_{..} + \theta_{P.} + \theta_{.nP} + \theta_{PnP} = 1$$

Equilibrium fraction of SUs in the processing state:

$$\theta^*_{PnP} = \left(\dot{k}^{-1} + \left(y_{EX_P}j_{X_P}\right)^{-1} + \left(y_{EX_{nP}}j_{X_{nP}}\right)^{-1} - \left(y_{EX_P}j_{X_P} + y_{EX_{nP}}j_{X_{nP}}\right)^{-1}\right)^{-1}$$

Figure S1: The interaction of the protein and non-protein substrates processed in parallel to produce the generalized reserves, E, and faeces, P.

We associate the arrival fluxes $j_{X_P}$ and $j_{X_{nP}}$ with the digestion rate, $J_d$. Thus, $j_{X_P} = a_P\dot{b}M_X$ and $j_{X_{nP}} = (1 - a_P)\dot{b}M_X$, where the association rate $\dot{b} = \{\dot{j}_{Xgm}\}L^2$. We link the association rate $\dot{b}$ with the maximum digestion rate and the dissociation rate $\dot{k}$ with the maximum specific assimilation rate, $\dot{j}_{EA_m}$, both relate to the surface area of the contact between stomach and its content $A_g$, which is taken proportional to $L^2$. Thus, $\dot{b} = \{\dot{j}_{Xgm}\}L^2$ and $\dot{k} = \{\dot{j}^d_{EA_m}\}L^2$, where $\{\dot{j}_{Xgm}\}$ and $\{\dot{j}^d_{EA_m}\}$ are the surface-specific maximum digestion and assimilation rates, respectively.

The rate of reserve formation or assimilation rate, $\dot{j}_{EA}$ (mol/d), equals

$$\dot{j}_{EA} = \dot{j}^d_{EA_m}\frac{M_X}{M_X + \frac{\{\dot{j}_{EA_m}\}}{\{\dot{j}_{Xgm}\}}\left(\left(y_{EX_P}a_P\right)^{-1} + \left(y_{EX_{nP}}(1-a_P)\right)^{-1} - \left(y_{EX_P}a_P + y_{EX_{nP}}(1-a_P)\right)^{-1}\right)} \quad \text{(S2)}$$

which can be written as

$$\dot{j}_{EA} = \{\dot{j}^d_{EA_m}\}f_X L^2 \quad \text{(S3)}$$

where $f_X$ represents the scaled functional response for digestion given by

$$f_X = \frac{M_X}{M_X + M^X_K} \quad \text{(S4)}$$

and $M^X_K$ the half saturation constant given by

$$M^X_K = \frac{\{\dot{j}^d_{EA_m}\}}{\{\dot{j}_{Xgm}\}}\left(\left(y_{EX_P}a_P\right)^{-1} + \left(y_{EX_{nP}}(1 - a_P)\right)^{-1} - \left(y_{EX_P}a_P + y_{EX_{nP}}(1 - a_P)\right)^{-1}\right) \quad \text{(S5)}$$

*S.3. Derivation of chemical indices*

The chemical indices express the relative abundance of hydrogen (*H*), oxygen (*O*) and nitrogen (*N*) relative to carbon (*C*). The chemical indices can be calculated when the proximate composition of feed, structure, and reserve in terms of macronutrients such as protein (*Pr*), lipids (*L*) and carbohydrates (*CH*) as well as the elemental composition of these macronutrients is known. We here assumed a fixed elemental composition in terms of carbon, hydrogen, oxygen and nitrogen ($n_{C*}$, $n_{H*}$, $n_{O*}$, $n_{N*}$) for the various macronutrients (where * could be *Pr*, *L* or *Ch*). The values (table) were calculated by obtaining the amino and fatty acid profiles of feeds as well as fish of various sizes, life stages, nutritional conditions and geographical origins and then using the molecular formulas of their constituent monomers to construct typical animal proteins, lipids and carbohydrates.

Given the proximate composition (*Pr, L, Ch*) of an organic compound, the chemical indices can be calculated using the equations in Table S3. In addition, the gross energy (*GE*) of those compounds is given by:

$$GE = (23.6Pr + 39.5L + 17.2Ch) \qquad \text{(S6)}$$

where 23.6, 39.5 and 17.2 kJ g$^{-1}$ are the combustible energy contents of crude *Pr*, *L* and *Ch*, respectively, as typically used in fish research and provided by the National Research Council (2011) (read in Schrama (Schrama et al., 2018)). Finally, if the Apparent Digestibility Coefficients (ADC) of macronutrients in the food ($\kappa_{X_P}, \kappa_{X_L}, \kappa_{X_{Ch}}$) are also known, then it follows that the digestibility of GE will be:

$$\kappa_{X_{GE}} = \frac{23.6\, Pr\ \ \kappa_{X_P} + 39.5\, L\, \kappa_{X_L} + 17.2\, Ch\, \kappa_{X_{Ch}}}{GE} \qquad \text{(S7)}$$

While for the digestibility of dry matter, the inorganic food components, namely the ash content (*A*) and its digestibility ($\kappa_{X_A}$) will also partake in the calculation:

$$\kappa_{X_{DM}} = \frac{Pr\ \ x_P + L\, \kappa_{X_L} + Ch\, \kappa_{X_{Ch}} + A\, \kappa_{X_A}}{1000} \qquad \text{(S8)}$$

The chemical potential of food ($\mu_X$) and fecal waste ($\mu_P$) are, respectively,

$$\mu_X = 1000\, GE\, w_X \qquad \text{(S9)}$$

and

$$\mu_P = 1000\left(1 - \kappa_{X_{GE}}\right) GE \frac{w_P}{1 - \kappa_{X_{DM}}} \qquad \text{(S10)}$$

where $w_X$ and $w_P$ are the molecular weights of food and faeces, respectively, and calculated as $w_X = 12n_{CX} + 1n_{HX} + 16n_{OX} + 14n_{NX}$ and $w_P = 12n_{CP} + 1n_{HP} + 16n_{OP} + 14n_{NP}$. The chemical indices are food type dependent, and their calculation is given by the equations in Table

S3. The gross energy (*GE*) and its digestibility $\kappa_{X_{GE}}$ are calculated in eq. S6 and S7, while the digestibility of dry matter $\kappa_{X_{DM}}$ in eq. S8.

Table S3: The chemical indices of the organic compound * (food, structural biomass, reserve, or faeces) calculated from the three macronutrients (protein (*Pr*), lipids (*L*) and carbohydrates (*Ch*)).

The chemical indices of the organic compound * (food, structural biomass, reserve, or faeces)

$$n_{O*} = \frac{12(n_{OPr}\theta_{Pr} + n_{OL}\theta_L + n_{OCh}\theta_{Ch})}{16(n_{CPr}\theta_{Pr} + n_{CL}\theta_L + n_{CCh}\theta_{Ch})}$$

$$n_{H*} = \frac{12(n_{HPr}\theta_{Pr} + n_{HL}\theta_L + n_{HCh}\theta_{Ch})}{(n_{CPr}\theta_{Pr} + n_{CL}\theta_L + n_{CCh}\theta_{Ch})}$$

$$n_{N*} = \frac{12n_{NPr}\theta_{Pr}}{14(n_{CPr}\theta_{Pr} + n_{CL}\theta_L + n_{CCh}\theta_{Ch})}$$

The chemical indices of the macronutrients # (*Pr*, *L*, *Ch*)

| | $\boldsymbol{n_{C\#}}$ | $\boldsymbol{n_{O\#}}$ | $\boldsymbol{n_{H\#}}$ | $\boldsymbol{n_{N\#}}$ |
|---|---|---|---|---|
| *Pr* | 44.9 | 30.4 | 7.6 | 16.2 |
| *L* | 76.7 | 11.4 | 11.5 | - |
| *Ch* | 44.4 | 49.4 | 6.2 | - |
